# A Smart Architecture for Diabetic Patient Monitoring Using Machine Learning Algorithms

**DOI:** 10.3390/healthcare8030348

**Published:** 2020-09-19

**Authors:** Amine Rghioui, Jaime Lloret, Sandra Sendra, Abdelmajid Oumnad

**Affiliations:** 1 Research Team in Smart Communications-ERSC–Research Centre E3S, EMI, Mohammed V University in Rabat, Rabat 10000, Morocco; aoumnad@emi.ac.ma; 2 Integrated Management Coastal Research Institute, Universitat Politecnica de Valencia, 46370 Valencia, Spain; sansenco@posgrado.upv.es

**Keywords:** internet of Things, diabetic patient monitoring, machine learning, data classification, healthcare

## Abstract

Continuous monitoring of diabetic patients improves their quality of life. The use of multiple technologies such as the Internet of Things (IoT), embedded systems, communication technologies, artificial intelligence, and smart devices can reduce the economic costs of the healthcare system. Different communication technologies have made it possible to provide personalized and remote health services. In order to respond to the needs of future intelligent e-health applications, we are called to develop intelligent healthcare systems and expand the number of applications connected to the network. Therefore, the 5G network should support intelligent healthcare applications, to meet some important requirements such as high bandwidth and high energy efficiency. This article presents an intelligent architecture for monitoring diabetic patients by using machine learning algorithms. The architecture elements included smart devices, sensors, and smartphones to collect measurements from the body. The intelligent system collected the data received from the patient, and performed data classification using machine learning in order to make a diagnosis. The proposed prediction system was evaluated by several machine learning algorithms, and the simulation results demonstrated that the sequential minimal optimization (SMO) algorithm gives superior classification accuracy, sensitivity, and precision compared to other algorithms.

## 1. Introduction

The field of healthcare is always evolving and offers many research possibilities. This evolution is based on the use of technologies and applications of the Internet of Things (IoT). It combines the information and communication technologies (ICTs), the use of sensors, the generation of massive data and the application of big data, machine learning techniques, and artificial intelligence. The use of new technologies is mainly used for the continuous monitoring of patients suffering from chronic illnesses [1], whose number has increased in recent years. Thus, IoT technology provides new solutions for diabetic patients.

Chronic diseases are characterized for having long duration and requiring long-term treatments. Patients with chronic diseases usually spend long periods in the hospital to be daily monitored. Some common chronic diseases can be heart disease, cancer, or diabetes. Currently, diabetic disease is very dangerous since it yearly generates the death of many people. Therefore, the diabetic patient needs to be controlled to lead a normal daily life.

Diabetes is a chronic disease related to a dysfunction of the pancreas that occurs when that organ does not produce the correct level of insulin or the body does not use the insulin properly [1]. High or low blood sugar levels can cause dysfunction and deterioration of many organs such as the eyes, the nerves, and the blood vessels. Therefore, a continuous and daily monitoring is required to avoid the worsening of the diabetic patient’s health.

In the last few years, the increase in the number of diabetic patients has required the use of more systems for monitoring these patients. Monitoring systems for diabetic patients are aimed at periodically monitoring blood glucose levels. As a result, patients, relatives, and doctors can follow the glucose readings at all times and quickly react when there is an abnormal reading.

Portable monitoring devices for monitoring diabetic patients offer several advantages including the improvement of the quality of life of diabetic patients by reducing hospitalization time. For this reason, the use of a wireless technology with very good coverage that allows sending data from patients to physicians is highly interesting. In this sense, 5th Generation (5G) technology, known as the next generation of mobile networks, permits high-speed transmission, bigger network capacity, and network scalability. However, the evaluation of this technology is currently focused on increasing the data transfer rate [2].

In this paper, we developed an architecture for the smart continuous monitoring of diabetic patients using a machine learning algorithm for data classification. Our proposed architecture included portable sensors to detect the blood sugar level, temperature, and physical activity of the patient. A smartphone transmitted the data collected by the sensors to a database station through the 5G cellular network. The collected and analyzed data included classification and prediction using several classification algorithms. Additionally, the proposed system helped diabetic patients to obtain future predictions of their blood sugar levels. The tests were performed using naïve Bayes, random forest (RF), ZeroR, simple logistic, sequential minimal optimization (SMO), and J48 as classification algorithms to process the diabetes dataset and determine which algorithm was the most powerful in determining the patient’s level of risk.

It is possible to use deterministic mathematical models for the treatment of diabetes. However, there have been few studies so far in the literature on mathematical models of diabetes mellitus. Stochastic numerical analysis remains an interesting method to study the epidemic disease tendency of diabetes mellitus [3,4]. In our proposed solution, we used a simple sensor with low power and low-cost solutions for the glucose monitoring application to generate data for classification. The patient’s data were updated in the cloud every day. Doctors used the collected data to monitor the patient’s blood glucose variation and to give the necessary medical care in the case of an incorrect glucose level. The prediction was reached by using several machine learning algorithms. In order to provide the best accuracy, different classification algorithms were analyzed, tested, and compared using different parameters.

The rest of the paper is organized as follows. Section 2 includes the related work. Section 3 presents the proposed architecture for diabetic patient monitoring using 5G. Section 4 describes the system implementation. Section 5 provides a brief description of data collection and Section 6 presents the results and discussion. Finally, conclusions and future work are outlined in Section 7.

## 2. Related Work

In this section, we present a summary of some published previous work related to 5G-based systems for blood sugar level monitoring in diabetic patients. The section also includes some existing works focused on big data and predictive analytics in healthcare that use classification to predict possible episodes of rises or falls in the blood sugar level. Classification in e-health monitoring plays a vital role in the further treatment of the disease.

A. Ahad et al. [5] present a review of 5G technology and IoT-enabled smart healthcare applications. The authors also state the challenges, research trends, and future research directions in the field of healthcare over 5G.

J. Lloret et al. [6] present an architecture and protocol for smart continuous e-health monitoring using 5G. The proposal is based on the use of a 5G smartphone and wearable devices to collect patients’ vital signs. The collected data are stored in a database and, using big data and machine learning techniques, the data are processed to create intelligent responses to send an alarm when the system detects an anomalous event.

In [7], M. Chen et al. propose a mobile health system using 5G for constant assessment and monitoring of diabetes patients. First, the authors present the 5G-Smart Diabetes system combining existing technologies such as Wearable 2.0, machine learning, and big data for creating comprehensive monitoring and analysis for diabetic patients. Subsequently, the authors show the data-sharing mechanism and the data analysis model for 5G-Smart Diabetes. Finally, the authors carried out a 5G-Smart Diabetes test bed. The results show that the system is able to provide personalized diagnosis and treatment for patients.

Another system based on the use of IoT is the indoor anti-collision alarm system (IAAS) presented by F. Xiao et al. [8]. The system, based on Radio Frequency Identification (RFID), is able to identify and track passive RFID tags by analyzing the received backscatter signals. The authors extracted the received signal strength indicator (RSSI) based on the log-normal distance pass loss (LWLR) algorithm and phase profiles as fingerprints to help blind users to avoid obstacles. Experiments showed that system’s good results with an accuracy of 94% in obstacle avoidance.

A. Goyal et al. [9] propose a smart home health monitoring system for predicting type 2 diabetes and hypertension. The goal of this system is to analyze the patient’s blood pressure and glucose readings at home. The caregiver is notified in case of any abnormality detected. The system also uses supervised machine learning classification algorithms to predict hypertension and diabetes status.

In [10], I. A. Najm et al. propose a new machine learning model based on a decision tree (DT) algorithm to predict the optimal enhancement of congestion control in the wireless sensors of 5G IoT networks. This model aims to determine the optimal parametric setting in a 5G environment.

H.B. Ahmed et al. [11] propose a new system for predicting the glucose concentration of diabetic patients. The authors use GlucoSim software to analyze patients’ information. In this system, the continuous glucose monitoring sensor (CGS) and the Kalman filter (KF) are used to reduce noise. This system helps to avoid the hypo- or hyperglycemia of serious complications.

K. Kannadasan et al. [12] aim to classify the Pima Indians diabetes dataset with better accuracy and other evaluation metrics. The authors propose a deep neural network framework for diabetes data classification using stacked autoencoders. The experiments are performed by using precision, recall, specificity, and F1 score as evaluation metrics for evaluating the system.

In [13], L. Wang et al. introduce an ensemble learning algorithm, XGBoost, to predict the risk of type 2 diabetes and compare their algorithm with support vector machines (SVMs), the random forest (RF) algorithm, and the k-nearest neighbor (KNN) algorithm in order to improve the prediction effect of existing models.

A. Charleonnan et al. [14] propose the use of certain machine learning techniques such as k-nearest neighbors (KNN), support vector machines (SVMs), logistic regression (LR), and decision tree classifiers for predicting chronic kidney disease using clinical data. In order to select the best technique for predicting chronic kidney disease, the authors compare the performance of these models.

In order to process the real-time accumulated biosensor input data, H. Yoo et al. [15] provide a model for personalized heart condition classification in combination with a fast and effective preprocessing technique and a deep neural network.

S. González-Valenzuela et al. [16] present a healthcare monitoring system based on a handoff protocol for continuous monitoring of ambulatory patients at home. This system is based on a two-tier network architecture, where one creates a layer of wearable sensors for vital sign collection and that the other creates a point-to-point link between the body sensor network coordinator device and an access point (AP). The experiments show packet loss rate down to 20% of the value otherwise obtained when solely using the point-to-point, coordinator–AP link, the best results were obtained when the sensor is placed on the wrist and the patient walks at a rate of 0.5 m/s.

Finally, we analyze different works focused on the use of machine learning algorithms. In [17], I. Izonin presents two methods for solving the classification task of medical implant materials based on the compatible use of the Wiener polynomial and SVMs. The author compares the proposed methods with existing algorithms.

In [18], T.L. Tepla et al. develop a classification method based on the application of multiclass logistic regression for the design of biocompatible materials in medical products in order to reduce the probability of incorrect alloy identification. R. Tkachenko et al. [19] compare the results of solving data classification problems using the most common classification methods and describe a new classification method based on neural-like structures of the geometric transformation model.

The main purpose of our work was to develop a new architecture for monitoring diabetic patients using 5G technology. None of the reviewed works focuses on the use of machine learning algorithms for classifying data from diabetic patient with a system based on 5G technology. The next section presents our proposal. Our system is not limited to monitoring this disease, although we focused it on the application of several machine learning algorithms to classify the data from diabetic patients and the parameters related to this disease.

## 3. Proposed Architecture

This section presents a detailed description of the proposed 5G architecture for a diabetic patient monitoring system. The aim of this paper was to monitor the blood glucose level of the diabetic patient using 5G technology to send the data and artificial intelligence in order to process the information and generate intelligent decisions.

The architecture of our smart system for continuous monitoring diabetic patients over 5G technology was composed of a set of sensors, wearable devices, an application running in a smartphone, and a server with a database. Several wireless technologies were used in the architecture. On the one hand, Wi-Fi was used to connect the different sensors to the smartphone. On the other hand, 5G technology was used to connect the smartphones to the cellular network for sending the data to the database server.

The proposed system was set up to collect data on the blood glucose level of diabetic patients, temperature, and physical activity and then transfer the data with the smartphone via the 5G connection to a base station. Afterward, using artificial intelligence and machine learning methods, the system intelligently processed the data to help users to control their glucose levels and predict future changes in health.

A diabetic patient requires continuous monitoring of their blood glucose level since, in a normal situation, small changes in glucose level do not indicate a problem for the patient’s health; however, continuous variations can imply very serious consequences such as diabetic coma, blindness, and even death. It is well known that diabetic patients generally follow a diabetes management plan by taking their insulin regularly. For this reason, we proposed a system for remotely monitoring the blood glucose level at home and being able to have a rapid intervention in case of medical emergency. Doctors would receive an alert message when a patient registered incorrect values. From that information, the physicians could recommend specific actions to treat the problem.

In 5G networks, cells are denser and smaller and should provide a very high transmission rate to network users. In the development of 5G networks, many types of devices exist with different characteristics and needs. The intelligent healthcare field requires a wide variety of sensors and devices, which generate various kinds of data that require the use of 5G technology. The correct processing and use of these data require different types of network functionality, such as mobility, charging, security, policy control, reliability, and latency to better manage healthcare solutions. 5G technology has the ability to manage health problems and reduce medical costs, keeping patients in touch with their doctors. It allows doctors to take care of their patients, wherever they are without the need to see them physically. 5G will be used for many health applications that require high bandwidth and reliable connectivity. The use of a 5G network will help doctors to quickly transmit massive reliable data files. Here are some advantages of 5G technology in comparison with 4G technology:

- High radio speed up to 20 Gbps.
- Low latency: this allows an autonomous driving, remote-controlled machine.
- Massive device connectivity: connection of IoT devices.
- Low battery consumption: 5G improves battery life by recovering energy from climate energy.

4G technology provides services such as Internet access with global roaming and full support for all other multimedia applications, whereas 5G technology offers a higher bandwidth with excellent device connectivity, a very high system capacity and energy conservation, with cost minimization. Table 1 shows the comparison between 4G and 5G technologies.

In our proposed system, we used 5G technology for remotely monitoring the patients since a 5G-based network has the capacity to support more than 60,000 connections with a very low latency.

**Figure 1:**
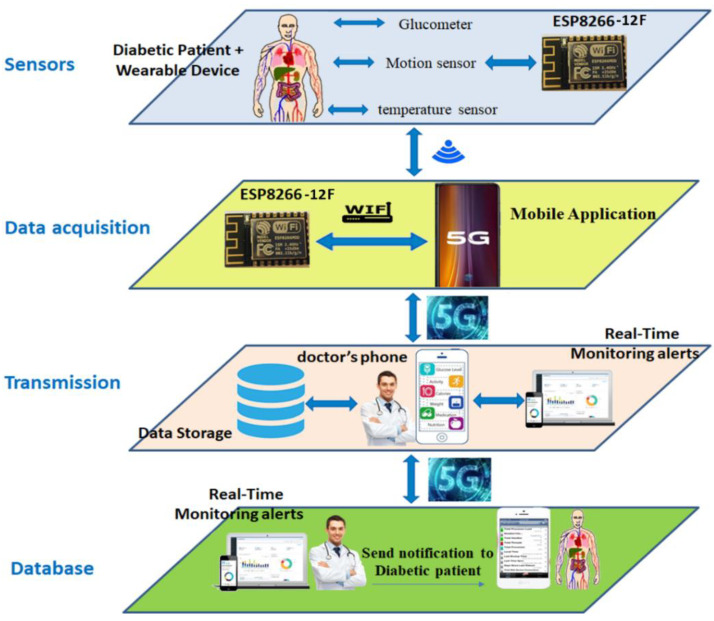
Proposed architecture for diabetic patient monitoring.

Our proposed architecture consisted of the following four main layers: (i) sensors part, (ii) data acquisition part, (iii) transmission part, and (iv) database part. Figure 1 presents our proposed architecture for monitoring diabetic patients.

- Sensors: this layer contains the blood glucose level sensor, temperature sensor, and motion sensor. This layer also contains the ESP8266 module that connects the sensors and gives them a wireless interface for sending the data to the patient’s smartphone. Therefore, the sensors are responsible for acquiring the data and transmitting it to the patient’s smartphone.
- Data acquisition layer: this part contains the patient’s smartphone and the application to collect data. Data from the sensors are displayed on the mobile application. Data are also sent to the base station via the 5G network, which allows a large number of simultaneous connections per covered area. Ultimately, it targets up to a million devices per km, i.e., ten times more than 4G.
- Transmission layer: the smartphone sends the data to the database using 5G for processing, and sends the data to the doctor’s phone for examination.
- Database layer: this is a processing unit that stores data from sensors to be processed and classified using several artificial intelligence algorithms. Using machine learning algorithms, the server decides whether the data collected are positive data (true positive (TP)) or negative data (false negative (FN)). When the system detects an abnormal situation, a notification is generated. The server sends a message to the doctor. The doctor checks the notification and sends their advice and treatments, which are displayed on the patient’s smartphone.

Smart healthcare is linked to a variety of short- and long-range communication technologies to transport data between devices and servers [20]. However, in order to transport data between sensors and the base station in intelligent healthcare, it is highly recommended to use long-range communication technologies such as General Packet Radio Service (GPRS) and mobile communication (GSM), Long-Term Evolution (LTE) or LTE Advanced, among others. For this study, we proposed to work with 5G networks to meet the needs of IoT devices in cellular networks and to improve battery life and coverage.

## 4. Hardware Design and Implementation

This section shows the proposed design of our diabetic patient monitoring system.

**Figure 2:**
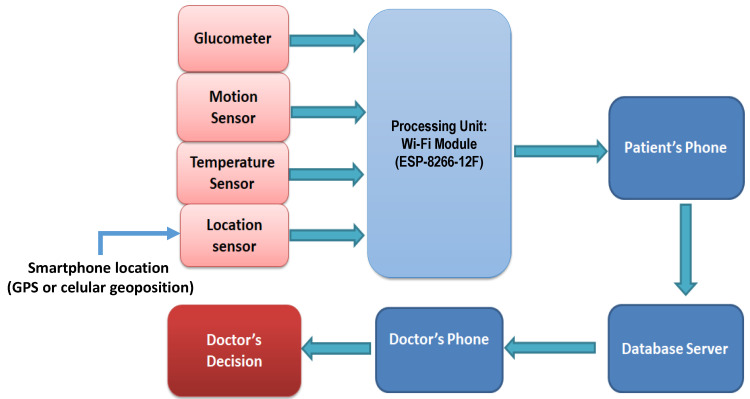
Hardware block diagram.

The developed device measured body temperature, physical activity of patient, blood glucose level, and oxygen saturation in the blood using various sensors. These parameters were transmitted using the ESP8266-12F module (Ai-Thinker, Shenzhen, China) to the smartphone. The measured values were analyzed and stored in a server in order to have them accessible for the patient’s and doctor’s smartphones. Finally, data were sent to medical experts to examine them. Figure 2 shows the block diagram of the proposed diabetic patient monitoring system.

### 4.1. ESP8266-12F Module

The system was based on the ESP8266 system on a chip (SoC). This module has been designed to cover the needs of a connected world. It is the core of the NodeMCU [21] and Wemos D1 Mini [22] Cards. The ESP8266 integrates a powerful processor with a 32-bit architecture as well as a clock frequency of 80MHz/160MHz and Wi-Fi connectivity. It offers a complete and self-contained Wi-Fi networking solution compatible with the IEEE 802.11 b/g/n standard. The module is powered by 3.3 V. It contains 17GPIO and 2 UART ports. The ESP-12F module has 4 MB of external flash memory and 32 kB of RAM memory. The node can be used to establish a Wi-Fi direct connection (P2P) or as a soft AP configuring it with AT commands.

### 4.2. Sensor

The blood glucose level was measured using a digital glucose sensor that also accurately measured the blood pressure. The body temperature could be calculated by putting the sensor in contact with the body. In order to measure motion, a pedometer was used that monitored the physical activity, while the location was extracted directly from the smartphone through the developed application. The data collected by the sensors were processed by the ESP8266-12F module.

**Figure 3:**
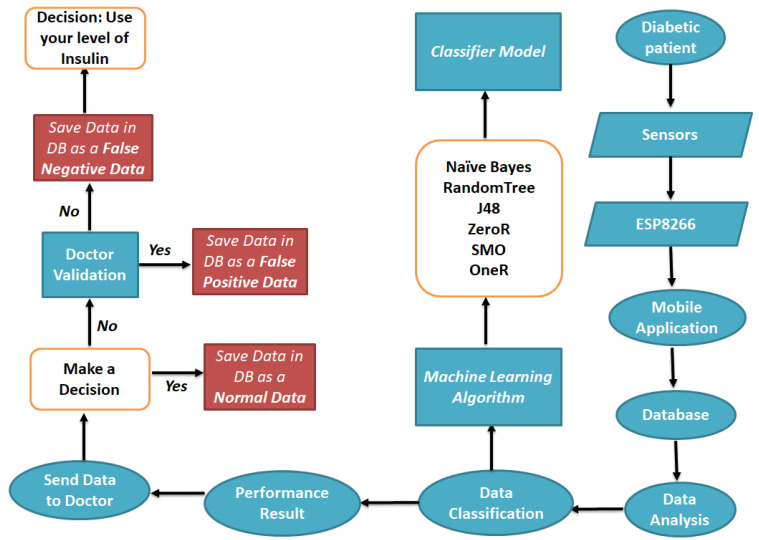
Proposed methodology flowchart.

Figure 3 presents the flowchart of the proposed system applied to perform the data classification in order to choose the best algorithm for building a classification model to predict possible episodes in diabetic patients.

To determine the blood glucose level, the diabetic patient took a drop of blood at the end of a finger and then introduced the sample into a glucometer at least three times a day. For the implementation of our application, we found it very difficult to perform the practical test on the diabetic patient. For this reason, we proposed to work with an SHT 31 temperature and humidity sensor that had the same connections. The glucometer was easy to use with an I2C connection, which was connected to the ESP8266-12F module to ensure the sending of data to the database. The ESP8266-12F module performed the processing as well as the WI-FI activities but with low energy consumption. The data collected by the sensors were sent to the ESP8266-12F, which were then analyzed and processed by the on-board ESP8266-12F microcontroller using the code loaded in its flash memory. The ESP8266-12F module connected to the Wi-Fi router, which worked as a station node, and transmitted data from the live sensor to the Internet database at regular intervals. This means that the ESP8266-12F plays the role of a sensor interface unit and the role of a wireless communication unit in addition to doing all of the processing.

**Figure 4:**
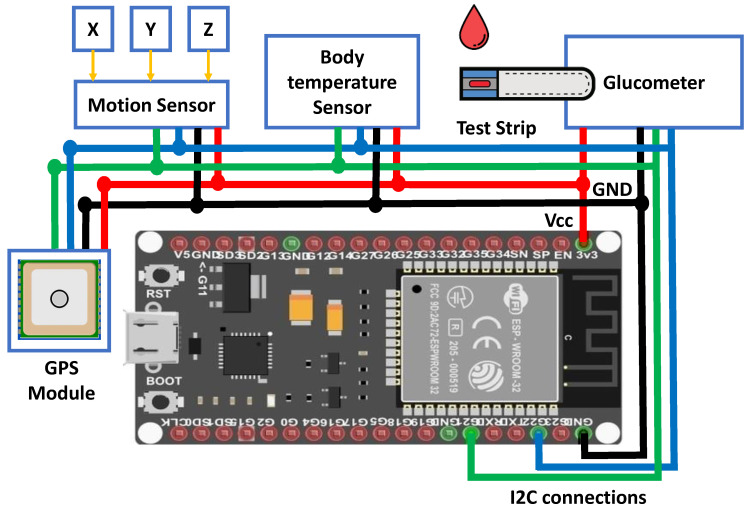
Connection of the ESP8266 module with the SHT 31 sensor.

The hardware implementation of the system is illustrated in Figure 4.

**Figure 5:**
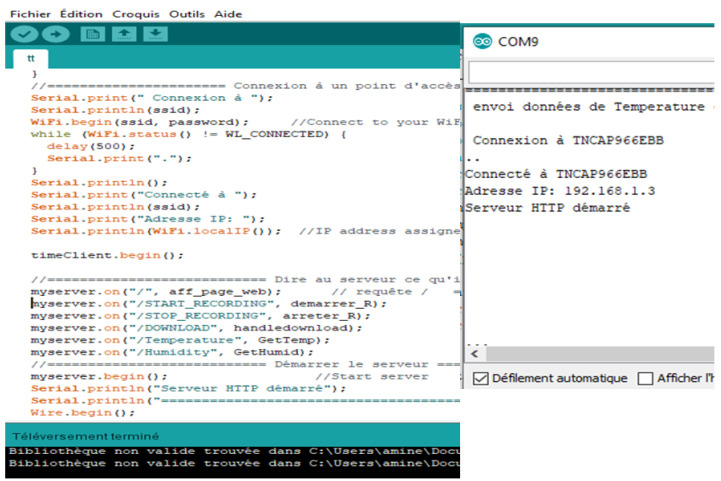
The Arduino Program of SHT 31 and Serial Monitor of Arduino IDE.

The ESP8266-12F acted as a server and we communicated with it as a client through a web page from a computer or smartphone. Figure 5 shows an example of Arduino code that contains the code to control the SHT31 connected to the ESP8266-12F module. The serial monitor shows the IP address of that module as well as the SSID of the network created.

**Figure 6:**
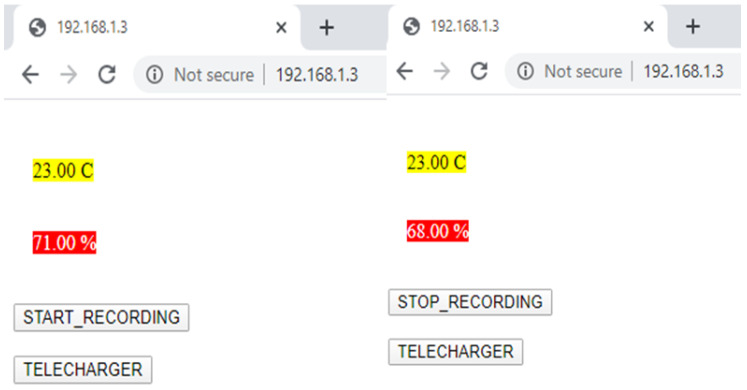
The SHT31 Sensor’s web Page.

The ESP8266-12F, configured as an access point, started the webserver and communicated its IP address to us. When we typed the IP address on a browser, it gave us a web page that contained Temperature and Humidity icons. The web page loaded these files automatically without updating the content of the field. In addition, a button (Start/Stop) was configured to save the measured (temperature and humidity, as an example) data in a *.csv file and a Download button to download the values saved in an Excel file for processing. Figure 6 shows an example of the web page created to the SHT31 sensor.

**Figure 7:**
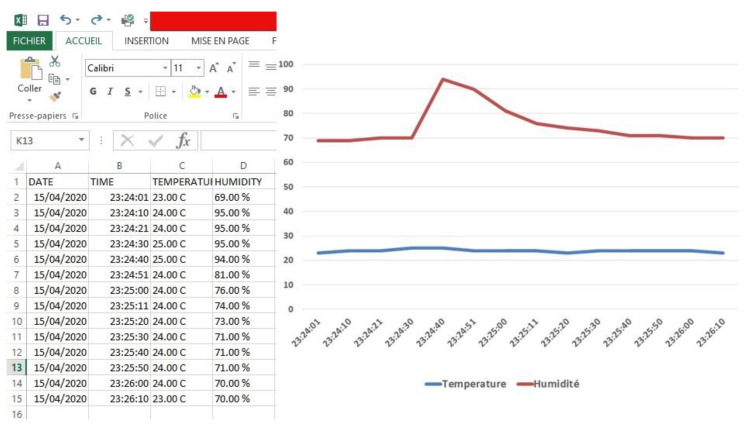
Data storage.

Therefore, if we clicked on the Download button, an Excel file was downloaded to our computer. This file contained the temperature and humidity values with the date and time when the data were collected, as shown in Figure 7. The final version contained the parameters useful for our goal.

## 5. Data Collection

This section shows the fundamental and the most important step of machine learning algorithms to collect and process the data. Table 2 presents the dataset used. The dataset used a database containing data on several diabetic patients. We used this dataset to try the different machine learning algorithms to detect and make predictions of diabetes. The dataset included the following attributes: gender, age, day the measures were taken, blood glucose level, insulin used, body temperature, and physical activity.

In this experiment, we used several automatic learning methods to choose the most adequate algorithm to make predictions in diabetic patients. The classification methods used in this experiment were naïve Bayes, J48, sequential minimal optimization (SMO), ZeroR, OneR, simple logistic, and random forest. The experiments were carried out using Weka software (Version 3, Waikato, New Zeland). The dataset used to perform the tests consisted of 10,807 data values.

Data classification performance (see Equation (1)) is measured by accuracy, sensitivity, specificity, and precision. We define accuracy using the following equation:

(1) $$\mathrm{Accuracy}\;=\;\frac{\mathrm{TP}+\mathrm{TN}}{\mathrm{TP}+\mathrm{TN}+\mathrm{FP}+\mathrm{FN}}\;(\%)$$

where TP is the value of true positive rate, TN is the value of true negative rate, FN is the value of false negative rate, and FP is the value of false positive rate.

Precision is estimated as the ratio between the value of true positives and the sum of the values of true positives and false positives (see Equation (2)).

Precision = TP/(TP + FP)(2)

Specificity (see Equation (3)) is defined as the ratio between the value of true negatives and the sum of the total value of true negatives and false positives.

Specificity = TN/(TN + FP)(3)

Sensitivity (see Equation (4)) is defined as the ratio between the value of true positives and the sum of the total value of true positives and false negatives.

Sensitivity = TP/(TP + FN)(4)

Recall is defined as the ratio between the value of false negatives and the sum of the total value of true positives and false negatives (see Equation (5)).

Recall = FN/(TP + FN)(5)

Finally, F-measure is a combination of precision and recall, and it is defined by the following equation (see Equation (6)):

(6) $$F-\mathrm{Measure}\;=\;\frac{2\times \mathrm{Precision}\times \mathrm{Recall}}{\mathrm{Precision}+\mathrm{Recall}}$$

## 6. Results and Discussion

This section presents the performance results in terms of precision, receiver operating characteristics (ROC), and accuracy as well as the discussion results.

Table 3 presents the accuracy level and the training time of the different algorithms used, i.e., naive Bayes, support vector machine, random forest, and simple classification and regression tree (CART) algorithm.

**Figure 8:**
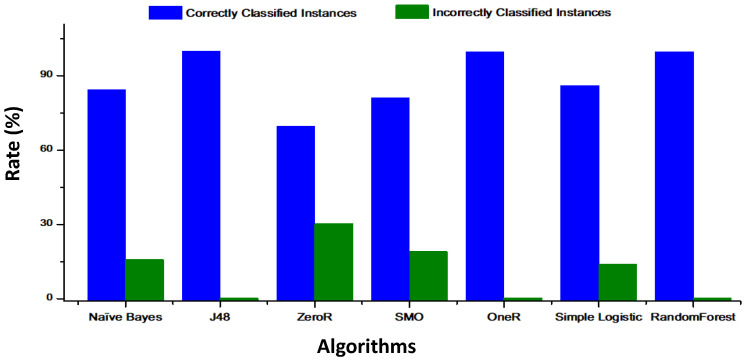
Rate of correctly and incorrectly classified instances of the algorithms.

Figure 8 shows a comparison between naïve Bayes, J48, ZeroR, SMO, random forest, simple logistic, and OneR in terms of the rate of correctly classified instances and incorrectly classified instances.

**Figure 9:**
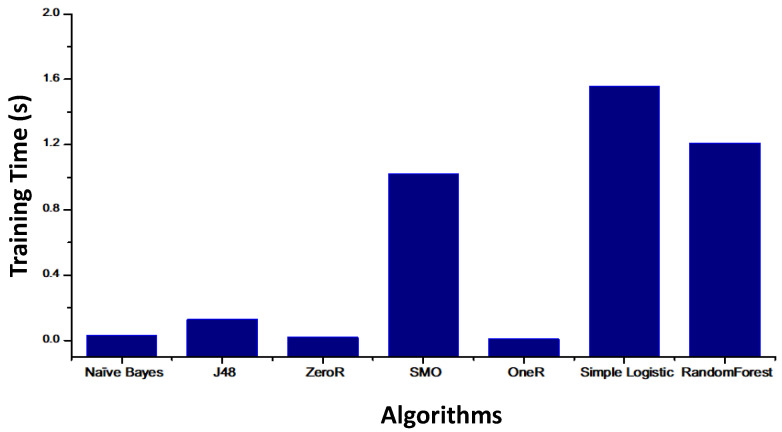
Training time results for the different algorithms.

Figure 9 shows the training time of each classification algorithm. The simple logistic takes the longest time (1.56 s), whereas the one that takes the shortest time is OneR, with a time about 0.01 s.

Table 4 shows different parameters such as TP rate and FP rate, precision, recall, and F-measure calculated from our results.

**Figure 10:**
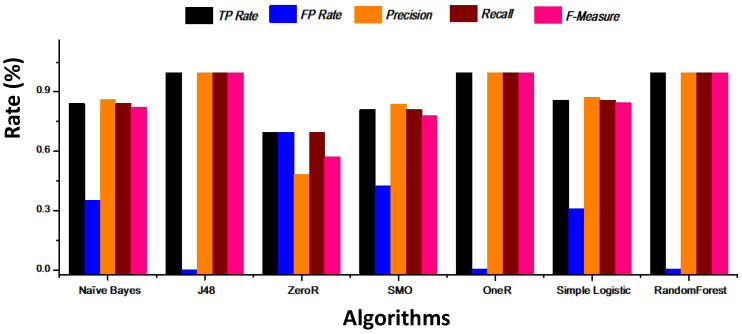
Performance results of TP, FP, precision, recall, and F-measure of the different algorithms.

Figure 10 show the performance results of each classifier in terms of precision, recall, and F-measure.

Table 5 shows the values of specificity, sensitivity, accuracy, and precision for the algorithms used in this study.

**Figure 11:**
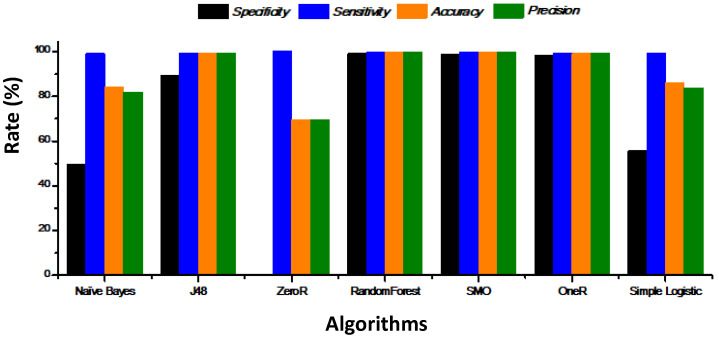
Specificity, sensitivity, accuracy, and precision of the different algorithms.

Figure 11 indicates the specificity, sensitivity, accuracy, and precision of the different algorithms. With respect to specificity, sensitivity, accuracy, and precision, OneR, RandomForest, and SMO are the algorithms that reach the best results.

Table 6 summarizes the result of Mean Absolute Error (MAE), Mean Squared Error (MSE), and Cohen’s Kappa for all tested algorithms in this study.

**Figure 12:**
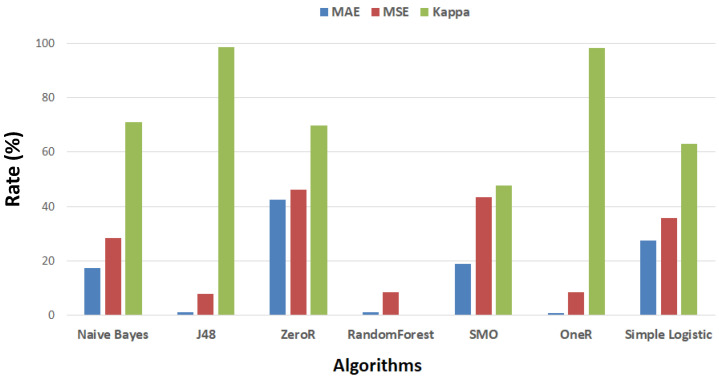
Rates of MAE, MSE, and Kappa for all algorithms.

The results presented in Figure 12 show that random forest is the most robust algorithm compared to the other algorithms.

We compared the details for different classification algorithms. We can observe in Table 4 that the precision obtained by SMO (99.66%) is better than the precision obtained by naïve Bayes, simple logistic, J48, OneR, and ZeroR. It is also easy to see that random forest presents the highest value of correctly classified instances with a training time of 1.21 s (see Table 2). After choosing random forest as the predicted model, we could now analyze the results obtained by evaluating the efficiency of our algorithms. In fact, Table 3 shows that random forest and J48 obtained the highest value (99%) of TP. The J48 and random forest algorithms present the lowest FP rate. From these results, we can conclude that random forest has outperformed the other classifiers.

## 7. Conclusions

Predictive analytics in healthcare can help doctors and medical researchers to obtain information from medical data and make intelligent and efficient decisions. For this study, we proposed a monitoring system for diabetic patients using 5G technology and machine learning algorithms. We created an intelligent algorithm based on artificial intelligence on big data capable of analyzing the data of diabetic patients and sending a notification in case of emergency. For this study, we employed a classification of diabetic patients using the WEKA tool according to six classifiers based on machine learning algorithms, i.e., naïve Bayes, J48, ZeroR, SMO, OneR, random forest, and simple logistic. These algorithms were compared in terms of precision and accuracy. The proposed system was evaluated by several machine learning algorithms (naïve Bayes, SMO, J48, ZeroR, OneR, simple logistic, and random forest) and the simulation results demonstrated that the SMO algorithm exhibited excellent classification with the highest accuracy of 99.66%, a sensitivity of 99.85%, and a precision of 99.66%.

For future work, we will examine the classification of each patient by adding other health parameters that should be taken into account to better measure the diabetes. Specifically, it could be interesting to add a galvanic skin response (GSR) sensor because when a person is suffering (or minutes before) a problem due to the low or high blood glucose level, usually experiment sweating. So, it could be a good indicator to predict episodes of hyperglycemia and hypoglycemia. A thermocouple could also be stuck to the skin to better measure the body temperature. Moreover, we plan to simulate sending massive data with 5G technology. Finally, we will work with other mathematical approaches and use new algorithms to improve the obtained results.

## Figures and Tables

**Table 1:** Comparison between 4G and 5G technologies.

Technology	4G	5G
Year of Appearance	2003	2020
Bandwidth	1 Gpbs	Higher than 1 Gbps
Technology	WiMAX, LTE, and LTE Advanced	Massive Multiple*-*input Multiple*-*output (MIMO), ultra-dense network, millimeter-wave technology, etc.
Throughput	100–300 Mbps	1–10 Gbps
Core Network	Internet	Internet
Service	All IP-based network service	High speed and capacity, large broadcasting of data in Gpbs
Web Standards	IPv4	IPv6
Processing	Multimedia services, high data rate	Increased flexibility, resource sharing, lower battery consumption

**Table 2:** Glucose level, temperature, and physical activity.

Day	Blood Sugar Level (mg/dL)			Temperature	Number of Steps
	Morning	Afternoon	Evening		
Day1	98	102	111	37	5423
Day2	166	153	124	36	6322
Day3	103	112	114	37	4876
Day4	134	102	98	37	4657
Day5	161	72	88	38	8511
Day6	150	147	123	36	4690
Day7	69	78	82	38	8768
Day8	100	104	111	37	4121
Day9	98	87	86	37	7823
Day10	61	70	77	38	8543

**Table 3:** Accuracy level and training time of the algorithms.

Algorithms	Correctly Classified Instances	Incorrectly Classified Instances	Training Time (s)
Naïve Bayes	84.1369%	15.8631%	0.03
J48	99.7619%	0.2381%	0.13
SMO	80.9524%	19.0476%	1.02
ZeroR	69.6875%	30.3125%	0.02
OneR	99.5685%	0.4315%	0.01
Simple Logistic	85.9077%	14.0923%	1.56
Random Forest	99.6577%	0.3423%	1.21

**Table 4:** Values of true positive (TP), false positive (FP), precision, recall, and F-measure for the algorithms.

Algorithms	TP Rate	FP Rate	Precision	Recall	F-Measure
Naïve Bayes	0.841	0.353	0.862	0.841	0.824
J48	0.998	0.004	0.998	0.998	0.998
SMO	0.810	0.426	0.838	0.810	0.781
ZeroR	0.697	0.69	0.486	0.697	0.572
OneR	0.996	0.008	0.996	0.996	0.996
Simple Logistic	0.859	0.312	0.875	0.859	0.846
Random Forest	0.997	0.006	0.997	0.997	0.997

**Table 5:** Values of specificity, sensitivity, accuracy, and precision for the algorithms.

Algorithms	Specificity	Sensitivity	Accuracy	Precision
Naïve Bayes	49.78%	99.08%	84.14%	81.94%
J48	89.49%	99.47%	99.17%	99.32%
SMO	98.92%	99.85%	99.66%	99.66%
ZeroR	0%	100%	69.69%	69.69%
OneR	98.32%	99.47%	99.11%	99.25%
Simple Logistic	92.32%	99.47%	99.11%	99.25%
Random Forest	55.67%	99.06%	85.91%	83.71%

**Table 6:** Values of mean absolute error (MAE), mean squared error (MSE), and Kappa for all algorithms.

Algorithms	MAE	MSE	Kappa
Naïve Bayes	17.5%	28.38%	70.89%
J48	1.2%	7.93%	98.53%
SMO	18.75%	43.3%	47.85%
ZeroR	42.65%	46.18%	69.69%
OneR	0.75%	8.63%	98.25%
Simple Logistic	27.49%	35.64%	62.89%
Random Forest	1.08%	8.56%	97.79%
